# Khoikhoi perspectives on public health: Indigenous values for a COVID-19 response in South Africa

**DOI:** 10.7189/jogh.11.03032

**Published:** 2021-03-01

**Authors:** Cornelius Ewuoso, Luis Cordeiro-Rodrigues

**Affiliations:** 1 Department of Medicine, University of Cape Town, South Africa; 2 Department of Philosophy, Yuelu Academy, Hunan University, China

The majority of literature on African public health ethics and bioethics is focused on values that come from the Bantu sub-Saharan majority [1]. Nevertheless, the Khoikhoi are also another important set of ethnic groups who have a distinctive value system. The Khoikhoi – once predominantly hunter-gatherers and nomadic farmers – are a group present in Southern African countries (eg, Botswana, Namibia, Angola, Zimbabwe, South Africa) who have a complete ethical system that includes dealing with health issues [2]. The Khoikhoi tend to live separately from the rest of the country and can be considered autonomous communities. This viewpoint explains the relevance of some of their distinctive views on key values that are relevant for addressing the COVID-19 pandemic in the context of South Africa. Notably, there are three core values from the Khoikhoi which are relevant for public health policies; these are Khoikhoi’s perspectives on i) distributive justice, ii) their views on the interconnectedness of individuals and iii) and their take on duties and entitlements according to age. This viewpoint argues that the Khoikhoi ideas offer useful insights for resource allocation, confinement policies, vaccination and triaging strategies during the pandemic.

## KHOIKHOI DISTRIBUTIVE JUSTICE

The Khoikhoi have a well-formed theory of distributive justice that is, contrasting with the Bantu theories, also commutative and legal. Distributive justice roughly describes what society owes individuals; commutative justice describes what individuals owe each other, and legal justice describes what individuals owe the society. The Khoikhoi only consume what they strictly need, and if they have excesses, they consider themselves to have the duty to share with others [2]. For example, the Khoikhoi generally do not hunt more than what they need to keep a balance with nature and with other communities. Furthermore, when the Khoikhoi hunt a large animal which is more than they can eat, they consider that it is their duty to share rather than stock the food. Finally, the Khoikhoi also contend that the worst-off need to be given special attention in the allocation of resources [2]. This principle can provide useful public health norms for addressing pandemic-hoarding and protecting the vulnerable. Particularly, it proscribes pandemic-hoarding that leaves others with very little to purchase; and if one has the economic and distributive capacity to allocate resources to others, one ought to do so. When the World Health Organization (WHO) declared COVID-19 a public health emergency on January 30, 2020, there was a race to the supermarkets and pharmacies by the general population to buy food, hygiene products, masks, nitrile gloves, alcohol-based disinfectants and other products in excess, which led some individuals having difficulties in buying products for their basic needs. At the beginning of the lockdown procedures in South Africa, for example, panic-buying significantly disrupted the food market and pushed up prices [3].

**Figure:**
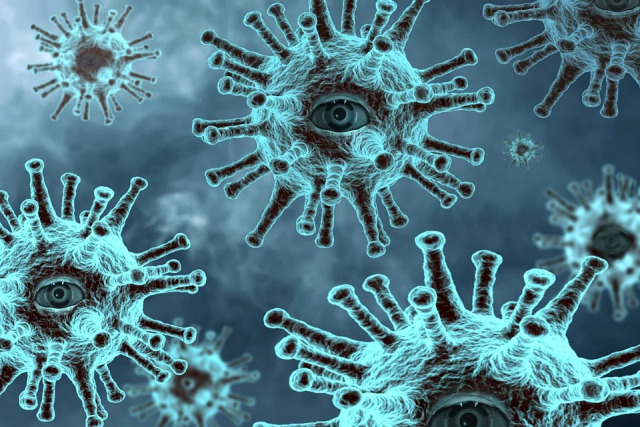
Photo: Coronavirus epidemic (from https://pxhere.com/en/photo/1608792).

The Khoikhoi principle of distributive justice suggests that hoarding and excessive consumption are immoral. One ought to buy only what one needs and never to the extent that others cannot acquire the products. The preceding implies that governments ought to impose limits, at least during a pandemic, on how many essential products each family unit can purchase, thus, ensuring that essential products are fairly distributed. Emergencies require special measures that might appear authoritarian in normal circumstances. Nevertheless, some of these measures may be temporarily necessary; in fact, most countries have been imposing such restrictions on movements and personal freedoms to curtail the spread of the virus. From a Khoikhoi perspective, it is not unreasonable to ration the supply of essential goods or impose restrictive measures. On top of this, when referring to essential goods, this kind of limitation does not seem to be unreasonable. Imagine if a rich person were to buy all the drinkable water in the world, this would impose an unreasonable cost on others. Justifiably this person should not be allowed to do so.

The Khoikhoi principle of distributive justice obliges the government to rectify unacceptable distribution of essential goods and resources amongst the population. This might require sharing of resources based on the principle of fundamental need; that is, prioritizing individuals such as the poor, whose survival depends on the distribution. This is especially the case because the pandemic has provoked a significant economic crisis and a higher number of people are in the line of poverty [4]. Specifically, in South Africa, many individuals survive on the daily income, which implies that with the lockdown measures, they have lost most of their already low income [5]. The way to rectify these injustices can vary, but some examples of rectifying distributive patterns can be taken from the case of what some countries have done; taxation increase for the rich, prohibiting firing people during the pandemic, making health care free for everyone during the pandemic, including illegal immigrants and allocating economic help packages to the poor [6].

Indigent individuals need help now more than ever. As reports [5] have shown, the South African government has largely failed to help more than half of its adult population, who are living below the poverty line before the pandemic. The situation has been predicted to worsen during and after the pandemic. Accurately, the World Bank has predicted that about 45% of the country’s population will be pushed below the poverty line 2020/2021 [5]. As part of its COVID-19 responses, the South African government announced that it would be topping up social grants awarded to indigent citizens. However, a lot more, such as an ambitious commitment to rootout social inequalities, ought to be done to alleviate poverty, and cushion the effects of the pandemic on struggling and impoverished families.

It is worth mentioning here that Khoikhoi’s distributive philosophy can also provide important insights on how international relations during the pandemic ought to be carried out. Particularly, during the peak of the pandemic, various countries were competing for health resources, and in some cases, they have kept resources which were not theirs [7]. Moreover, there was very little solidarity amongst countries, mostly trying to monopolize vital medical resources [1]. The Khoikhoi philosophy of distribution requires a supportive form of cooperation, especially in times of scarcity like this one. Although our focus here is the South African context, note that this international cooperation is relevant here to the extent that to overcome the pandemic, there is a need for a holistic approach that takes into account the economic and health situation of all.

## INTERCONNECTION

Like the Bantu, the Khoikhoi believe that every individual forms a link in a web of life. However, the Khoikhoi go a step further to add that a wrong that happens to one individual is connected to a wrong that happens to another individual. For example, the Khoikhoi usually believe that when a man goes hunting and fails, this failure will also cause someone else to fail at some duty. For the Khoikhoi, there is, therefore, a causal connection between the wrongs that happen [2]. Surely, this kind of connection is not always true, and there are good reasons to be skeptical about it. Nevertheless, the principle of interconnection grounded in the Khoikhoi value system provides a useful guide to follow during the current pandemic.

More specifically, this idea of interconnectedness realizes that privileging some ethnicities or social classes, failing to implement restrictive measures of social distancing or mask-wearing, can lead to a rise in infection rate, thus, creating significant health challenges or an overload of health care systems, as it has happened during this pandemic. Put differently, the nature of the virus suggests that it is not possible to overcome the pandemic without a holistic approach that neither privileges certain social groups nor leaves marginalized people aside. If it does, the cycle of the pandemic starts again, and people get infected. Consequently, pandemic-related policies must be inclusive and should be enforced everywhere [1].

The South African government was one of the first countries in Southern Africa to announce 5-tiered restrictive measures, and nationwide lockdown (on March 27, 2020). Under level 5 of the 5-tiered South African lockdown measure, which started on March 27, all citizens were expected to limit their outdoor trips to only essential ones such as to the grocery or the hospital. On its part, the South African government mostly concentrated its efforts on enforcing these policies in urban areas, while largely neglecting townships and other suburban poor settlements by not only not properly enforcing the confinement but also not giving conditions for sanitation and social distancing in those areas. Hence, there was significant neglect of the poorest classes in the South African approach. Based on the indigenous Khoikhoi value system, the above are, roughly, failures to act in ways that can improve public health. Interconnection requires governments to take public health policies wholistically, ie, not privilege some groups over others and give each the resources and supervision they need to protect themselves from the virus.

## AGE-BASED DUTIES AND ENTITLEMENTS

In Khoikhoi societies, age plays a vital role in determining individuals’ duties and entitlements [8]. Leadership is most often a hereditary position that is passed on to the eldest son. Younger and healthy individuals have the duty to collect food; elder individuals have very few duties – such as mediating civil disputes – in the community. Contrary to most cultures below the Sahara, younger individuals are entitled to more resources and the elder to less. The rationale is that elders have already had the opportunity to live their lives, and now they should give the same opportunity to the young.

The preceding theory of entitlements, according to age, provides guidance to governments on confinement strategies, allocation of vaccines, medical attention and ventilators. In terms of confinement strategies, it suggests confinement by age; broadly speaking, it recommends to confine the elder as a risk group, but de-confine the younger. The South African 5-tiered confinement strategy mostly consists of restricting movements for everyone. But most young black individuals in South Africa, depend on the daily income for survival. A Khoikhoi-inspired plausible approach to prevent a dilemma between dying of hunger or dying of COVID-19 is to introduce age-based de-confinement. Particularly, because the younger have stronger duties, and play a vital role by working and thus, prevent economic collapse in the global South, the Khoikhoi prescription suggests that only the group at-risks (especially the elder) ought to be confined while others continue working.

Likewise, it suggests that the younger ought to be vaccinated first to protect the old for at least three reasons. First, the young have strong immune systems and therefore can react better to the vaccination side-effects, whose long-term (adverse) effects mostly remain unknown given the exceptional speed with which they have been developed. In normal circumstances, a vaccine would require several steps and could take many years to develop. However, the urgency of developing a COVID-19 vaccine has necessitated the need to conduct some of these steps in parallel, thus significantly reducing the duration of the COVID-19 vaccine development to only a few months while still enforcing strict safety measures [9]. Second, given that the younger are more exposed because of being de-confined, they can transport the virus and vaccinating them first may be a way to avoid the spread and protect vulnerable groups. Third, given that the economic impact of this current pandemic has been massive, prioritizing vaccines to the young will help boost the economy. The prescription we recommend differs slightly from President Cyril Ramaphosa’s vaccination plan that mainly consists of “prioritizing’ essential workers, the elderly and other vulnerable individuals [10].

The Khoikhoi offer a perspective, which considers that the young ought to be given priority in situations of medical resource scarcity (eg, priority in ventilator allocation). We, however, do not think this latter point about the allocation of medical resource would be easily implemented in Southern African countries. One important aspect of the success of a policy is that it has wide public approval, especially in countries where there is a strong civil society as South Africa that routinely contests measures [11]. Contrasting with other measures which resemble and are not too distant from what Sub-Saharan Africans believe, this latter measure sharply contrasts most African views on respecting elders and, therefore we anticipate that there would be an initial backlash against this policy.

## CONCLUSION

This viewpoint has explained the relevance of the underexplored Khoikhoi ethic for addressing the current COVID-19 pandemic looking at the case of South Africa. It argued that the Khoikhoi understanding of values of interconnection, distributive justice, and age-based duties and entitlements can offer an improved approach for addressing the current shortcomings of the South African approach to COVID-19.
